# SNR Degradation in Undersampled Phase Measurement Systems

**DOI:** 10.3390/s16101772

**Published:** 2016-10-24

**Authors:** David Salido-Monzú, Francisco J. Meca-Meca, Ernesto Martín-Gorostiza, José L. Lázaro-Galilea

**Affiliations:** 1 Institute of Geodesy and Photogrammetry, ETH Zürich, Zürich 8093, Switzerland; 2 Department of Electronics, University of Alcalá, Madrid 28871, Spain; meca@depeca.uah.es (F.J.M.-M.); ernesto@depeca.uah.es (E.M.-G.); lazaro@depeca.uah.es (J.L.L.-G.)

**Keywords:** phase measurement, undersampling, digitization, noise aliasing, optical ranging

## Abstract

A wide range of measuring applications rely on phase estimation on sinusoidal signals. These systems, where the estimation is mainly implemented in the digital domain, can generally benefit from the use of undersampling to reduce the digitizer and subsequent digital processing requirements. This may be crucial when the application characteristics necessarily imply a simple and inexpensive sensor. However, practical limitations related to the phase stability of the band-pass filter prior digitization establish restrictions to the reduction of noise bandwidth. Due to this, the undersampling intensity is practically defined by noise aliasing, taking into account the amount of signal-to-noise ratio (SNR) reduction caused by it considering the application accuracy requirements. This work analyzes the relationship between undersampling frequency and SNR reduction, conditioned by the stability requirements of the filter that defines the noise bandwidth before digitization. The effect of undersampling is quantified in a practical situation where phase differences are measured by in-phase and quadrature (I/Q) demodulation for an infrared ranging application.

## 1. Introduction

Estimating the phase difference between two sinusoidal signals is a typical technique in a wide range of measurement applications such as: specification and calibration of electronic and sensor systems [1,2], interferometric applications [3], impedance spectroscopy [4], optical [5] or ultrasonic [6] time-of-flight ranging, near-field direction-of-arrival estimation [7], laser anemometry [8] or radio frequency control in synchrotrons [9,10]. Several techniques can be used to estimate the phase difference, such as quadrature phase detectors [11] and lock-in amplifiers [12], sine wave fits [13], cross-correlation [14] and methods based on the discrete Fourier transform [15]. These techniques are mainly implemented in the digital domain, from the data obtained from an analog-to-digital (A/D) conversion system after some analog processing. The different methods differ in how they combine the requirements determined by the application, mainly being: computing load, observation time, sensitivity against additive noise and signal distortion, and the suitability of coherent or non-coherent sampling. However, regardless of the method, signal-to-noise ratio (SNR) of the processed data is a key factor to obtain high accuracy. This SNR depends on the original properties of the information and the effects suffered by it in the processing prior the estimation, more specifically: the frequency response of the analog channel and the features of the data acquisition system.

The useful information bandwidth in phase measuring applications, determined by the spectral distribution of the phase variations, is usually much smaller than the frequency of the sine wave carrying that information. This situation can benefit from the use of undersampling or bandpass sampling [16,17], allowing a reduction in the requirements of the acquisition system and subsequent digital processing. The cost of using undersampling is a reduction of SNR due to noise aliasing in those situations where noise cannot be sufficiently band-limited. Reducing requirements and processing complexity may be highly convenient (or necessary) in certain systems where the sensor needs to be as simple and inexpensive as possible. For instance, in the context of the practical application shown in this work (addressed at the end of the introduction), indoor positioning in intelligent spaces, where the receivers are deployed in a large positioning area, there is a high number of sensors. Consequently, reducing the complexity and cost of these sensors is necessary for the feasibility of such system.

From a conceptual point of view, strictly limiting noise bandwidth before undersampling, hence reducing noise aliasing, can be easily achieved by means of selective band-pass filtering. However, from a practical point of view, the inherent instabilities in the frequency response of any analog system causes uncertainty in the phase estimation. This limits the practical application of undersampling, specifically the selection of sampling frequency. This frequency does not depend on the information bandwidth but on the noise bandwidth, which is defined by the stability requirements of the analog system frequency response and the effect of noise aliasing on the SNR of the digitized signal.

The instability of an analog system frequency response is defined by the instability of the real parameters of its components. The main problem is caused by the variation of the operation temperature in relation to the temperature during the system calibration. Selective band-pass filters derive in high sensitivities to the real parameters of its components and the parasitic elements present in any electronic design. Consequently, undersampling frequency must be set after the design of the analog system and the definition of the noise spectrum, depending on the phase stability requirements and its effect on the frequency response.

This work aims at quantifying the degradation of the phase difference estimation in the discrete domain between two sinusoidal signals as a consequence of SNR reduction due to noise aliasing when undersampling is applied. The application context of this work is presented in Section 2, in order to study the effect of noise aliasing in a practical situation. In Section 3, the SNR degradation in the digitized signal as a function of the undersampling intensity is quantified, taking into account the practical limitations of the filtering before digitization. The particular method used for the phase difference measurement is explained in Section 4, and the expression of the standard deviation of the differential phase error as a function of the SNR calculated in Section 3 is derived. Finally, the study is particularized in Section 5 for a specific application where differential phase estimations are used for optical ranging, comparing the results with real measurements from the application set-up.

## 2. Context

The application that contextualizes this work and the particular method used to estimate the phase are described next in order to provide quantitative results on the effect of undersampling in a practical situation. However, the study developed in this work can be easily generalized to any application where digital phase estimation of sinusoidal signals is used, considering the specific definition of the filter before digitization and the translation between SNR and precision set by the particular phase estimation method.

This work has been developed in the context of a phase-shift measuring system for infrared distance estimation to be applied in indoor mobile robot positioning [18]. Indoor positioning systems rely, in most cases, on certain kind of distance estimation. The distance estimation can be based on several techniques, generally related with signal strength, normally from radiofrequency signals, or time of flight (TOF), typically applied to ultrasound [19,20], ultra-wideband or optical signals. However, most accurate distance meters are based on optical technology, which, on the other hand, has been hardly studied for indoor positioning applications. This motivated the development of the indoor positioning system contextualizing this work, where near infrared signals differential time of arrival is used.

There are basically two non-interferometric methods for estimating distance using optical signals with relatively high precision: direct TOF of short pulses [21] and TOF indirectly estimated from phase-of-arrival [22]. An interesting approach is described in [23], where the phase-based method, implementing a double frequency modulation, is applied for wide-range and high-resolution ranging using undersampling. The pulse-based technique is suitable for long range applications, allowing safe high-power emissions due to the short duration of the pulses. The phase-based method, though allowing shorter ranges, achieves higher precision due to the use of continuous information from the signal. For this reason, considering that distances in indoor environments are generally rather limited, phase estimation proved to be the most adequate option for an optical indoor positioning system.

This document focuses on one of the phase difference measuring units that form the system [24]. A series of photodiodes, placed on fixed and known positions in the environment will receive a 6 MHz sinusoidal intensity modulated infrared (IR) (940 nm) signal with different delays. This signal is continuously emitted in a wide pattern from the robot to position, whose light-emitting diode (LED) based source operates independently from the receivers, free of any synchronization mechanism with the rest of the system. The phase of this incoming signals, apart from an unknown offset common for all receivers, is proportional to the distance between the emitter and each detector. Every received signal is filtered and amplified by a low level conditioning stage and A/D converted by undersampling. The resulting digitized signal is then demodulated with two locally generated I/Q references and the phase-shift between the incoming signal and an equivalent one from another receiver is extracted from the resulting demodulated sequences.

**Figure 1:**
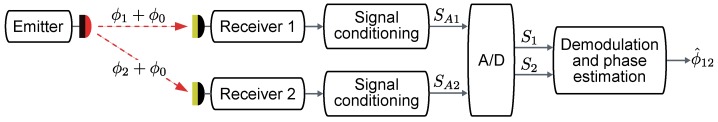
General structure of the differential phase estimator.

Figure 1 shows the general structure of one of the differential phase measuring units forming the system. $\phi_{1}+\phi_{0}$ and $\phi_{2}+\phi_{0}$ are the phases upon reception, where $\phi_{1}$ and $\phi_{2}$ are proportional to their respective propagation paths, and $\phi_{0}$ is the unknown initial phase of the emitter, common for all receivers hence canceled in the differential measurement. $S_{A1}$ and $S_{A2}$ are the input analog signals, $S_{1}$ and $S_{2}$ are the digital signals obtained after undersampling and ${\hat{\phi }}_{12}$ is the estimated differential phase between both signals.

## 3. Digitization

An analysis on the reduction of SNR in the band of interest due to noise aliasing during digitization as a function of sampling frequency is provided in this section. All the results are normalized to a given input SNR in the signal band and can be easily generalized to any input SNR.

### 3.1. Signal Conditioning

The conditioning stage of the system used for the analysis is formed by a single transimpedance amplifier that converts the generated photocurrent into a voltage plus an additional second order band-pass filter. This filtering stage is critical for the analysis presented in this document since it will define the noise bandwidth of the signals to be digitized, and the final performance defined by the sampling parameters will be strongly dependent on noise aliasing after the conversion and demodulation. As will be demonstrated in this section, a more restrictive anti-alias filtering of the analog signal would reduce these effects, but it would also add a higher phase uncertainty due to unwanted drifts of the components.

The generic transfer function of a second order band-pass filter is shown in Equation (1), where $f_{0}$ is the central frequency of the filter and *Q* is the quality factor, having unity gain at $f=f_{0}$:

(1) $$H\left(jf\right)=\frac{j\frac{1}{Q}\frac{f}{f_{0}}}{{\left(j\frac{f}{f_{0}}\right)}^{2}+j\frac{1}{Q}\frac{f}{f_{0}}+1}.$$

The phase introduced by the filter is

(2) $$\phi_{{}_{H\left(jf\right)}}=\left\{\begin{array}{cc} -\mathrm{atan}\left[Q\left(\frac{f}{f_{0}}-\frac{f_{0}}{f}\right)\right], & \;\;\mathrm{if}\;\;\;\;f_{0}>f,\\ \pi -\mathrm{atan}\left[Q\left(\frac{f}{f_{0}}-\frac{f_{0}}{f}\right)\right], & \;\;\mathrm{if}\;\;\;\;f_{0}<f. \end{array}\right.$$

The phase variation due to a change $\Delta f_{0}$ in $f_{0}$ is

(3) $${\left.\Delta \phi_{{}_{H\left(jf\right)}}\right|}_{{}_{\Delta f_{0}}}\approx \frac{\partial \phi_{{}_{H\left(jf\right)}}}{\partial f_{0}}\Delta f_{0}=\frac{Q}{1+{\left[Q\left(\frac{f}{f_{0}}-\frac{f_{0}}{f}\right)\right]}^{2}}\left(\frac{f}{f_{0}}+\frac{f_{0}}{f}\right)\frac{\Delta f_{0}}{f_{0}},$$

and the phase variation due to a change ΔQ in *Q* is

(4) $${\left.\Delta \phi_{{}_{H\left(jf\right)}}\right|}_{{}_{\Delta Q}}\approx \frac{\partial \phi_{{}_{H\left(jf\right)}}}{\partial Q}\Delta Q=\frac{-Q\left(\frac{f}{f_{0}}-\frac{f_{0}}{f}\right)}{1+{\left[Q\left(\frac{f}{f_{0}}-\frac{f_{0}}{f}\right)\right]}^{2}}\frac{\Delta Q}{Q}.$$

Assuming that $f\approx f_{0}$ and Q is small, Equation (3) can be simplified to

(5) $${\left.\Delta \phi_{{}_{H\left(jf\right)}}\right|}_{{}_{\Delta f_{0},f\approx f_{0}}}\approx 2Q\frac{\Delta f_{0}}{f_{0}}.$$

It can be observed that the phase sensitivity to variations in $f_{0}$ increases in a direct proportion to the quality factor of the filter.

Under the same assumptions, Equation (4) can be approximated to

(6) $${\left.\Delta \phi_{{}_{H\left(jf\right)}}\right|}_{{}_{\Delta Q,f\approx f_{0}}}\approx 2Q\frac{f_{0}-f}{f_{0}}\frac{\Delta Q}{Q},$$

resulting in the phase sensitivity to variations of the quality factor being directly proportional to the quality factor and the relative deviation of $f_{0}$ to the tone frequency. In a practical analysis, the latter sensitivity can be neglected compared with the one shown in Equation (5), given that $f\approx f_{0}$.

As can be deduced from the analysis, the sensitivity of the filter phase to variations in its parameters is directly proportional to its quality factor. This defines practical limits to its value and, consequently, to the noise spectrum reduction required for undersampling. Taking this into account, once the admissible phase uncertainty is defined for the particular application specifications, the design of the band-pass filter can be addressed, obtaining the relationships that define the minimum stability of its components and the maximum value of the quality factor. In the application proposed in this work, Q=0.7 is used, which allows limiting the phase error to ±1 mrad (±8 mm in ranging considering the propagation speed of the optical signal and the modulation frequency of 6 MHz) versus relative variations of $f_{0}$ up to ±715 ppm. The stability considered for $f_{0}$ can be achieved in a wide temperature range using components with an average temperature stability.

**Figure 2:**
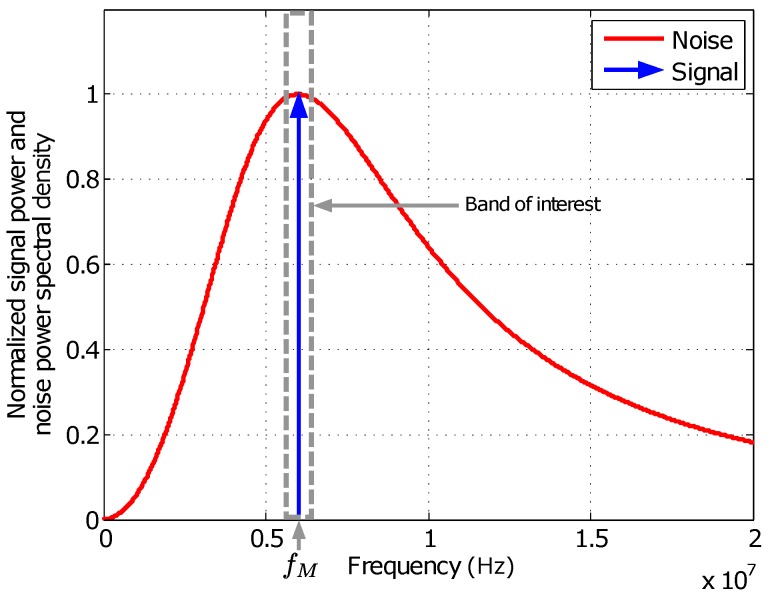
Normalized single-sided power spectral density of noise and signal power before sampling.

Considering the conditioning stage structure and a flat noise distribution with constant power density $N_{0}$ before the band-pass filter, which is an adequate approximation for the bandwidth of interest, the analog signal to be sampled will present the single-sided power spectral density shown in Figure 2, where both signal and noise are normalized to $A_{n}^{2}/4$ and $N_{0}$, respectively, where $A_{n}$ is the signal amplitude. All the information is contained in the phase of the modulated signal at $f_{M}$, while the noise power spectral distribution $N_{A}\left(f\right)$ is described by

(7) $$N_{A}\left(f\right)=N_{0}{\left|\frac{j\frac{1}{Q}\frac{f}{f_{0}}}{{\left(j\frac{f}{f_{0}}\right)}^{2}+j\frac{1}{Q}\frac{f}{f_{0}}+1}\right|}^{2},$$

where $f_{0}=f_{M}=6$ MHz and Q=0.7.

The signal-to-noise ratio, calculated for a reference bandwidth of 1 Hz, is defined for this analog signal as

(8) $${\mathrm{SNR}}_{0}=\frac{A_{n}^{2}/4}{N_{0}\cdot 1\mathrm{Hz}}.$$

This SNR will be used as a reference in the further analysis of the dependence of final SNR with sampling frequency, and adapted to any specific noise bandwidth when needed.

### 3.2. Sampling

The approach to reduce the sampling speed requirement of the analog-to-digital converter (ADC) is based on undersampling or band-pass sampling. The problem associated with band-pass sampling in the described system is related to noise aliasing due to aliasing phenomenon, which does not cause any loss of information but an increase of the noise power density in the band of interest. Since the analog noise bandwidth cannot be further reduced before sampling to keep an adequate phase stability, any sampling rate not far above $f_{M}$ will cause noise aliasing to some degree.

Note that, from now on, all the equations refer to discrete time signals, both for the noise aliasing and phase error analyses. This study bridges the analog input parameters with the final error in the estimation. However, the phase error study would be completely equivalent for a similar analog processing structure, adding the effects of the analog demodulation that can be neglected in its discrete time equivalent, like amplitude imbalance or quadrature error between different I/Q branches [25]. Considering this, continuous-time notation is used in all the document to provide an easier generalization of the development.

The total overlapped noise spectral density after the A/D conversion $N_{A/D}\left(f\right)$ is calculated from Equation (9), the sum of the infinite images of the original noise figure being $N_{A}\left(f\right)$, frequency shifted every integer multiple of the sampling frequency $f_{s}$:

(9) $$N_{A/D}\left(f\right)=\sum_{k=-\infty }^{+\infty }N_{A}\left(f-kf_{s}\right),$$

with k∈Z.

From the signal point of view, the subsequent phase estimation is carried out on the input alias $f_{M}^{{}^{\prime }}$ in the band $\left[-f_{s}/2,\;+f_{s}/2\right]$, calculated as

(10) $$f_{M}^{{}^{\prime }}=\left|f_{M}-kf_{s}\right|,$$

where *k* is the alias index that satisfies

(11) $$-\frac{f_{s}}{2}\leq f_{M}-kf_{s}<\frac{f_{s}}{2}$$

being

(12) $$\frac{f_{M}}{f_{s}}-\frac{1}{2}\leq k<\frac{f_{M}}{f_{s}}+\frac{1}{2}.$$

The alias used to carry out the measurement will present a phase-shift with respect to the original signal, but this shift is equal for every simultaneously sampled received signal, so this effect is canceled by the differential measurements and does not need to be considered.

Assuming again a reference bandwidth of 1 Hz, the signal-to-noise ratio for the digitized signals in the band of interest around the measuring alias frequency $f_{M}^{{}^{\prime }}$ is

(13) $${\mathrm{SNR}}_{A/D}=\frac{A_{n}^{2}/4}{N_{A/D}\left(f_{M}^{{}^{\prime }}\right)\cdot 1\mathrm{Hz}}.$$

**Figure 3:**
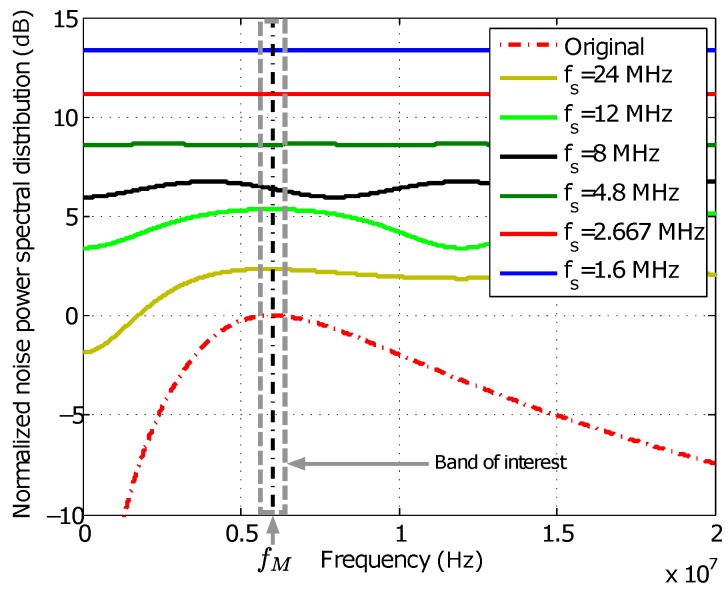
Computation of the normalized noise power spectral distribution after digitization for different sampling frequencies $\left(f_{s}\right)$.

Figure 3 shows the expected noise power spectral distribution $N_{A/D}\left(f\right)$, computed from the former expression, after digitizing with five different sampling frequencies. The resulting noise densities are normalized to the original density $N_{A}\left(f\right)$ in the analog signal evaluated at $f_{M}$. It can be seen how, due to the increment of noise aliasing, noise density is increased in the band of interest around the signal frequency when sampling frequency is reduced. The sampling frequencies shown in the figure are not arbitrary, but chosen to optimize the computing cost of the I/Q-based demodulation stage by reducing the complexity of its filters. The conditions to calculate this optimal frequencies, as well as the structure of the demodulation stage itself, will be explained in the phase estimation section.

**Figure 4:**
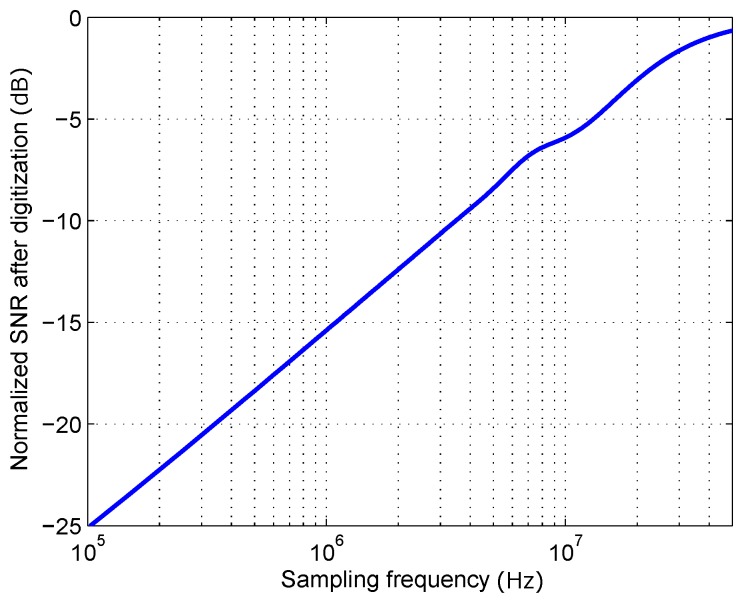
Computation of the normalized SNR after digitization as a function of sampling frequency $\left(f_{s}\right)$.

Figure 4 shows the dependence of ${\mathrm{SNR}}_{A/D}$ with sampling frequency. The result is normalized to the input SNR $\left({\mathrm{SNR}}_{0}\right)$. It can be seen that when sampling frequency gets higher, the amount of noise aliasing is reduced, and the digitized signal SNR tends to the original input SNR. The absence of hardly any noise power above 25 MHz can be seen in the leveling off of the signal degradation towards 0, showing how sampling above 50 MS/s would hardly cause any aliasing for the given noise spectrum.

## 4. Phase Estimation

The relationship between the uncertainty in the estimated differential phase and the SNR of the digitized signals depends on the particular method used to carry out the estimation. The applied method is defined in this section, allowing us to quantify, in terms of phase uncertainty, the effects of the SNR degradation due to undersampling.

### 4.1. Demodulation and Phase Measurement

The processing of the sequences obtained from the ADC is based on an asynchronous I/Q demodulator. The digital signal $S_{n}$ is multiplied by two locally generated in-phase and quadrature reference signals ($I_{ref},Q_{ref}$), shifted π/2 rad between each other and deviated Δf with respect to the expected frequency $f_{M}^{{}^{\prime }}$ of the input alias where the measurement will be carried out.

The resulting signals ($S_{nI},S_{nQ}$) will present a low frequency component around Δf and a high frequency component around $2f_{M}^{{}^{\prime }}$ plus Δf, which is digitally filtered. Finally, an arctangent estimator is applied to extract the phase from the low frequency components ($S_{nI}^{{}^{\prime }},S_{nQ}^{{}^{\prime }}$). The resulting phase ${\hat{\phi }}_{n}$ is not constant in static phase conditions due to the frequency difference between the incoming signal and the references, and should be subtracted with an equivalent phase obtained from another sensor, simultaneously acquired and demodulated. Finally, the resulting signal phase transitions are corrected, producing the final phase signal to be filtered in order to get every single phase-shift read-out.

**Figure 5:**
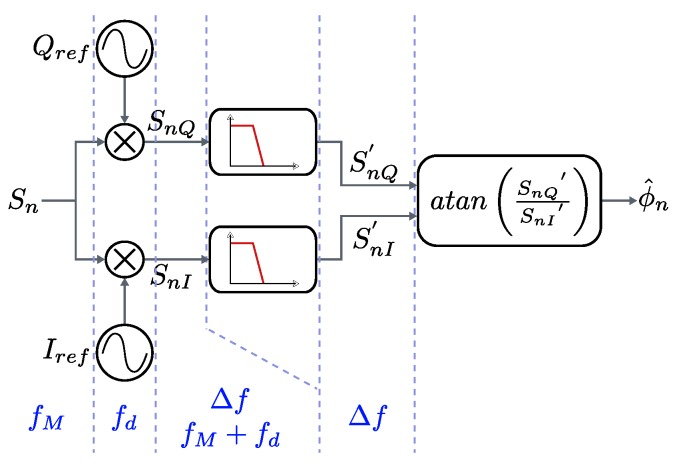
Phasemeter.

**Figure 6:**
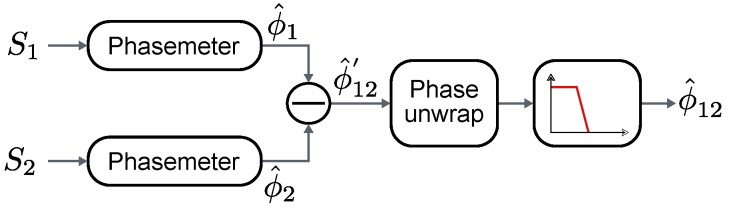
Differential distance estimation.

**Figure 7:**
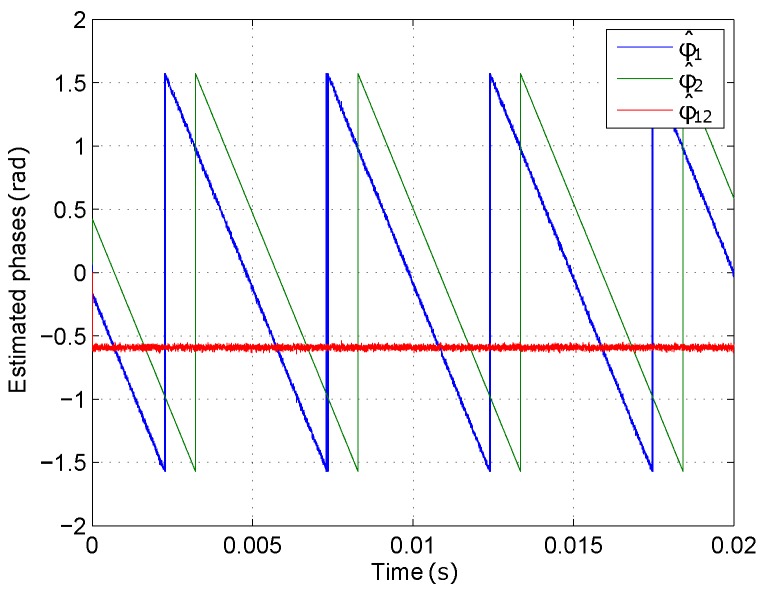
Output of phasemetters (${\hat{\phi }}_{1}$, ${\hat{\phi }}_{2}$, ${\hat{\phi }}_{12}$ (unwrapped)).

Figure 5 shows the phasemeter for every single incoming sequence, formed by the I/Q demodulator and the arctangent-based phase estimator. Figure 6 shows the differential phase computation and the final filtering stage. The outputs of two phasemeters, simultaneously processing the signals from two sensors, are shown in Figure 7. Note that both slopes, whose values depend on Δf, are equal for both signals, therefore a constant value ${\hat{\phi }}_{12}^{{}^{\prime }}$ is yielded by the subtraction, corresponding to the estimated relative phase difference between them.

**Figure 8:**
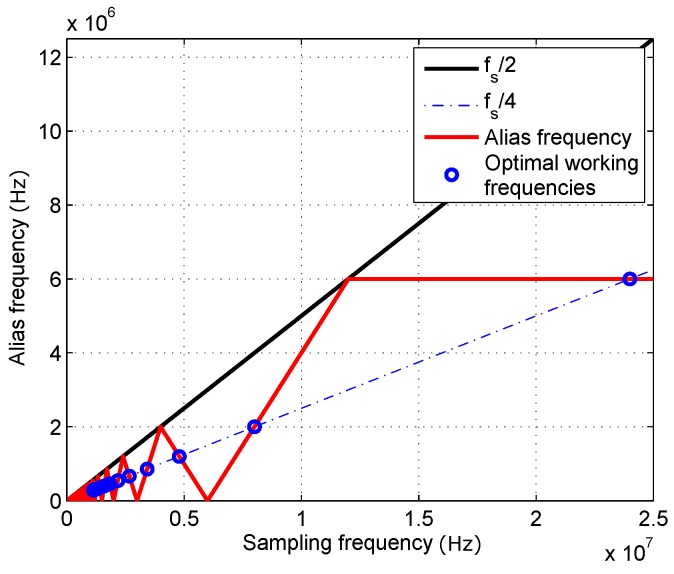
Alias frequency values $\left(f_{M}^{{}^{\prime }}\right)$ as a function of sampling frequency $\left(f_{s}\right)$. Optimal working frequencies.

Mixing in the demodulation stage splits the incoming signal into two spectral components with equal power, frequency shifted $+f_{d}$ and $-f_{d}$, respectively, where $f_{d}$ is the frequency of the reference signals used for demodulation. In order to guarantee that the high frequency component after the mixing is sufficiently attenuated by the digital filter in every I/Q branch, so its effect can be neglected, optimal working frequencies are defined so that the separation between both components is maximized. This allows, for a given necessary attenuation, reduction of the complexity of the digital filter, hence its computing cost. The frequency difference Δf between the modulation frequency of the input signal $f_{M}$ and the demodulation references frequency $f_{d}$ is defined as small as possible to maximize the separation between components. Considering this, the maximum separation condition implies using sampling frequencies so that the resulting alias $f_{M}^{{}^{\prime }}$ in the $\pm f_{s}/2$ band lies in $f_{s}/4$. This way, after the mixing, the low frequency component will be close to DC, and the high frequency component will be close to $f_{s}/2$, providing the maximum possible separation for a given sampling frequency. Possible alias frequencies as a function of sampling frequency are depicted in Figure 8, together with the sampling frequencies that fulfill the defined criterion.

A consideration has to be made for the definition of Δf. Applying the condition for choosing optimal sampling frequencies, explained in the previous paragraph, the low frequency component where the measurement is performed will ideally lie in Δf. Tolerances in the input signal frequency and sampling frequency due to clock inaccuracies have to be considered, so that the deviation from the ideal value does not cause the low frequency component to go below DC, avoiding phase inversions that should be corrected afterwards. This implies using a Δf as small as possible, but always higher than the possible frequency deviation in the resulting alias from its ideal value.

The low pass filter shown in Figure 6 is implemented as a moving average. This process, computed in a window of $T_{m}$ seconds, is modeled as a frequency response as follows

(14) $$H_{\mathrm{avg}}\left(f\right)=\frac{1}{T_{m}f_{s}}\cdot \frac{1-e^{-j2\pi fT_{m}}}{1-e^{-j2\pi f/f_{s}}},$$

where $T_{m}f_{s}$ is the number of samples of the averaging window.

The final noise equivalent bandwidth (NEBW), assuming a high number of samples in the averaging window ($T_{m}f_{s}>>1$), is defined by this filter as

(15) $$\mathrm{NEBW}=\int_{0}^{\frac{f_{s}}{2}}{\left|H_{\mathrm{avg}}\left(f\right)\right|}^{2}df=\frac{1}{2T_{m}}.$$

### 4.2. Phase Error Model

In this section, the model for the complete phase estimation process will be explained and developed so that an expression of the final phase error can be defined as a function of the signal and noise power levels in the input of the demodulation stage. This SNR (${\mathrm{SNR}}_{A/D}$) will be determined, as shown in previous sections, by the amount of noise aliasing defined by the band-pass sampling frequency.

Define the signal coming from receiver 1 after band-pass sampling as

(16) $$S_{1}\left(t\right)=A_{1}\sin\left(\omega_{M}t+\phi_{1}\right)+n_{1}\left(t\right),$$

where $\omega_{M}$ is $2\pi f_{M}$, $n_{1}\left(t\right)$ is the temporal expression of the overlapped noise after the digitization, shown in Equation (9) in the frequency domain, and $\phi_{1}$ is the single-phase true value for receiver 1 in static conditions.

The reference demodulation signals are

(17) $$\begin{array}{cc} I_{ref}\left(t\right) & =\cos\left(\omega_{d}t\right),\\ Q_{ref}\left(t\right) & =\sin\left(\omega_{d}t\right), \end{array}$$

where $\omega_{d}$ is $2\pi f_{d}=2\pi \left(f_{M}-\Delta f\right)$.

The signals after the mixing and filtering of the resulting high frequency components are

(18) $$\begin{array}{c} S_{1I}^{{}^{\prime }}\left(t\right)=\frac{A_{1}}{2}\cos\left[\left(\omega_{M}-\omega_{d}\right)t+\phi_{1}\right]+n_{1}^{{}^{\prime }}\left(t\right)\cos\left(\omega_{d}t\right),\\ S_{1Q}^{{}^{\prime }}\left(t\right)=\frac{A_{1}}{2}\sin\left[\left(\omega_{M}-\omega_{d}\right)t+\phi_{1}\right]+n_{1}^{{}^{\prime }}\left(t\right)\sin\left(\omega_{d}t\right), \end{array}$$

where $n_{1}^{{}^{\prime }}\left(t\right)$ is the equivalent noise signal after the digital low-pass filter.

Define noise signals normalized with their corresponding amplitude as

(19) $$n_{1N}^{{}^{\prime }}\left(t\right)=\frac{n_{1}^{{}^{\prime }}\left(t\right)}{A_{1}/2}$$

and form a signal composed by the I/Q components as

(20) $$D_{1}\left(t\right)=e^{-j\left(\Delta \omega t+\phi_{1}\right)}+n_{1N}^{{}^{\prime }}\left(t\right)e^{j\omega_{d}t},$$

where Δω=2πΔf, the differential phase for these composed signals coming from two receivers, would be

(21) $$\phi_{12}\left(t\right)=∠\left[D_{1}\left(t\right)\right]-∠\left[D_{2}\left(t\right)\right]=∠\left[\frac{D_{1}\left(t\right)}{D_{2}\left(t\right)}\right],$$

where ∠[·] is the argument operator.

Replace the signals by their definition

(22) $$\phi_{12}\left(t\right)=∠\left[\frac{e^{-j\left(\Delta \omega t+\phi_{1}\right)}+n_{1N}^{{}^{\prime }}\left(t\right)e^{j\omega_{d}t}}{e^{-j\left(\Delta \omega t+\phi_{2}\right)}+n_{2N}^{{}^{\prime }}\left(t\right)e^{j\omega_{d}t}}\right],$$

(23) $$\phi_{12}\left(t\right)=∠\left[e^{j\left(\phi_{2}-\phi_{1}\right)}\frac{1+n_{1N}^{{}^{\prime }}\left(t\right)e^{j\left[\left(\omega_{d}+\Delta \omega \right)t+\phi_{1}\right]}}{1+n_{2N}^{{}^{\prime }}\left(t\right)e^{j\left[\left(\omega_{d}+\Delta \omega \right)t+\phi_{2}\right]}}\right],$$

(24) $$\phi_{12}\left(t\right)=\phi_{2}-\phi_{1}+\Delta_{n_{1N}}\left(t\right)-\Delta_{n_{2N}}\left(t\right),$$

where

(25) $$\begin{array}{ccc} \Delta_{n_{1N}}\left(t\right) & = & ∠\left[1+n_{1N}^{{}^{\prime }}\left(t\right)e^{j\left[\left(\omega_{d}+\Delta \omega \right)t+\phi_{1}\right]}\right],\\ \Delta_{n_{2N}}\left(t\right) & = & ∠\left[1+n_{2N}^{{}^{\prime }}\left(t\right)e^{j\left[\left(\omega_{d}+\Delta \omega \right)t+\phi_{2}\right]}\right]. \end{array}$$

Developing the expression of the noise residuals for every receiver yields

(26) $$\Delta_{n_{1N}}\left(t\right)=\mathrm{atan}\left[\frac{n_{1N}^{{}^{\prime }}\left(t\right)\sin\left[\left(\omega_{d}+\Delta \omega \right)t+\phi_{1}\right]}{1+n_{1N}^{{}^{\prime }}\left(t\right)\cos\left[\left(\omega_{d}+\Delta \omega \right)t+\phi_{1}\right]}\right],$$

which, considering that noise instant values are much smaller than the unit, can be approximated to

(27) $$\Delta_{n_{1N}}\left(t\right)\approx \mathrm{atan}\left[n_{1N}^{{}^{\prime }}\left(t\right)\sin\left[\left(\omega_{d}+\Delta \omega \right)t+\phi_{1}\right]\right].$$

Considering the argument is very small, atan(x) can be approximated to *x* as

(28) $$\Delta_{n_{1N}}\left(t\right)\approx n_{1N}^{{}^{\prime }}\left(t\right)\sin\left[\left(\omega_{d}+\Delta \omega \right)t+\phi_{1}\right].$$

Applying the same development for the residuals from receiver 2 and averaging over $T_{m}>>\frac{2\pi }{\omega_{d}}$, the estimated phase is

(29) $${\hat{\phi }}_{12}\left(t\right)\approx \frac{1}{T_{m}}\int_{T_{m}}\left[\phi_{2}-\phi_{1}+n_{1N}^{{}^{\prime }}\left(t\right)\sin\left[\left(\omega_{d}+\Delta \omega \right)t+\phi_{1}\right]-n_{2N}^{{}^{\prime }}\left(t\right)\sin\left[\left(\omega_{d}+\Delta \omega \right)t+\phi_{2}\right]\right]dt.$$

The mean value of this estimated phase random variable is the actual phase difference $\left(\phi_{2}-\phi_{1}\right)$, and the estimation typical error, considering that both noise contributions are uncorrelated, is

(30) $$\sigma_{{\hat{\phi }}_{12}}\approx \sqrt{\frac{\sigma_{n_{1N}}^{2}}{2}+\frac{\sigma_{n_{2N}}^{2}}{2}},$$

where $\sigma_{n_{1N}}$ and $\sigma_{n_{2N}}$ are the standard deviations of noise after digitization, normalized with their corresponding signal amplitudes and integrated over the measurement bandwidth $T_{m}$. Undoing the normalization yields

(31) $$\sigma_{{\hat{\phi }}_{12}}\approx \sqrt{\frac{{\left(\frac{\sigma_{n_{1}}}{A_{1}/2}\right)}^{2}}{2}+\frac{{\left(\frac{\sigma_{n_{2}}}{A_{2}/2}\right)}^{2}}{2}}=\sqrt{\frac{2\sigma_{n_{1}}^{2}}{A_{1}^{2}}+\frac{2\sigma_{n_{2}}^{2}}{A_{2}^{2}}},$$

where $\sigma_{n_{1}}$ and $\sigma_{n_{2}}$ are the standard deviations of the voltage noise contained in the digitized signals prior demodulation, integrated in the measurement bandwidth. Considering the system’s speed constraints, the final averaging will be processed over a window in the milliseconds or tens of milliseconds range, providing an NEBW below 1 kHz in any case. Given such a narrow BW compared to the original noise bandwidth, noise density in the band of interest can be adequately approximated to a constant with value $N_{A/D}\left(f_{m}^{{}^{\prime }}\right)$. Considering this, the standard deviations of the voltage noises are calculated as

(32) $$\sigma_{n}=\sqrt{N_{A/D}\left(f_{m}^{{}^{\prime }}\right)\mathrm{NEBW}},$$

so the standard deviation of the final differential phase estimation is approximated to

(33) $$\sigma_{{\hat{\phi }}_{12}}\approx \sqrt{\mathrm{NEBW}\left(\frac{2N_{1_{A/D}}\left(f_{m}^{{}^{\prime }}\right)}{A_{1}^{2}}+\frac{2N_{2_{A/D}}\left(f_{m}^{{}^{\prime }}\right)}{A_{2}^{2}}\right)},$$

which, applying the definition in Equation (13), yields

(34) $$\sigma_{{\hat{\phi }}_{12}}\approx \sqrt{\frac{\mathrm{NEBW}}{2}\left(\frac{1}{{\mathrm{SNR}}_{1_{A/D}}}+\frac{1}{{\mathrm{SNR}}_{2_{A/D}}}\right)}.$$

It can be seen that the final approximation for the estimation typical error is related to the square root of the inverse of the combined SNR of both receivers in the signal band after digitization.

## 5. Results

**Figure 9:**
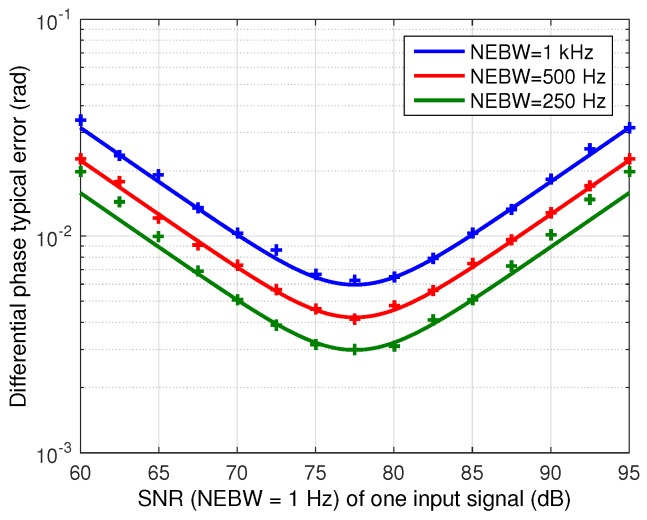
Phase typical error $\left(\sigma_{{\hat{\phi }}_{12}}\right)$ for different noise equivalent bandwidths as a function of SNR of one digitized input signal (${\mathrm{SNR}}_{1_{A/D}}=$[60 dB, 95 dB]) while the other input signal SNR is its complementary value (${\mathrm{SNR}}_{2_{A/D}}=$[95 dB, 60 dB]). Theoretical (solid) and simulations (crosses).

First, a validation of the phase error model, summarized in Equation (34), is provided. The theoretical expression is compared with measurements with the digital implementation of the phasemeter on a PC, where signals with different SNR levels, directly generated in the digital domain, are introduced. Figure 9 shows the validation results, where the theoretical and measured standard deviation of the differential phase error is shown as a function of one of the input signals SNR for different NEBW. The SNR of this input signal ${\mathrm{SNR}}_{1_{A/D}}$ takes values between 60 dB and 95 dB while the SNR of the other signal ${\mathrm{SNR}}_{2_{A/D}}$ is set to its complementary value in the same range, i.e., between 95 dB and 60 dB. The results show that the phase error model derived in Section 4.2 is a good approximation of the phase estimation behavior affected by noise.

**Figure 10:**
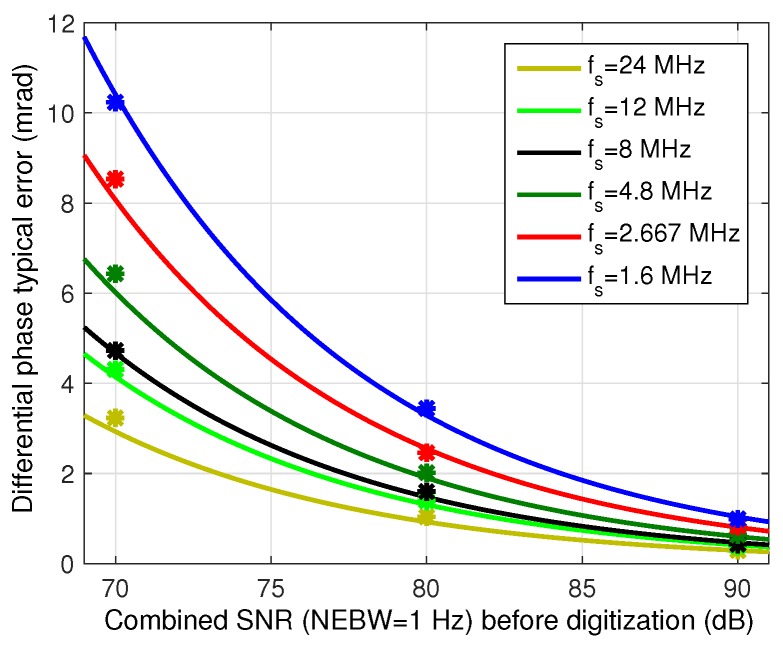
Phase error $\left(\sigma \right)$ as a function of input SNR $\left({\mathrm{SNR}}_{0}\right)$ and sampling frequency $\left(f_{s}\right)$. Estimated and measured results.

A computation of the expected phase error as a function of the original signal-to-noise ratio in the analog signal at 6 MHz $\left({\mathrm{SNR}}_{0}\right)$ and six different sampling rates is shown in Figure 10. Note that all the sampling rates used both in Figure 3 and Figure 10 fulfill the optimal working frequencies condition defined in the phase estimation section and depicted in Figure 8, excluding the 12 MHz case. This frequency has been added for comparative purposes, being the minimum sampling frequency that fulfills Nyquist theorem for a low-pass sampling approach. An averaging window of 20 ms has been selected to calculate the results. This value has been defined considering the maximum robot speed (0.5 m/s) and allowable error (±5 cm) of the positioning system this phasemeter has been designed for. This averaging window yields an NEBW of 25 Hz.

Note that $\left({\mathrm{SNR}}_{0}\right)$ in Figure 10 refers to the combined SNR of both input signals, calculated as ${\mathrm{SNR}}_{0}=\left({\mathrm{SNR}}_{01}{\mathrm{SNR}}_{02}\right)/\left({\mathrm{SNR}}_{01}+{\mathrm{SNR}}_{02}\right)$.

In order to validate the defined relationship between input SNR and phase error, measurements with real signals has been carried out, being the dots in Figure 10. The IR emitter and two of the receivers developed for the system described in [24] have been deployed so that the combined SNR of both received signals was 70, 80 and 90 dB. These signals have been acquired with different sampling frequencies in a 12-bit simultaneous-sampling digitizer, and processed using the previously explained demodulation architecture implemented on a PC using 20 ms averaging windows. The standard deviation of the final differential phase error has been calculated over 20 measurement windows for every position and averaged. It can be seen that the real results are very similar to the expected phase error obtained from the theoretical analysis for every frequency.

**Figure 11:**
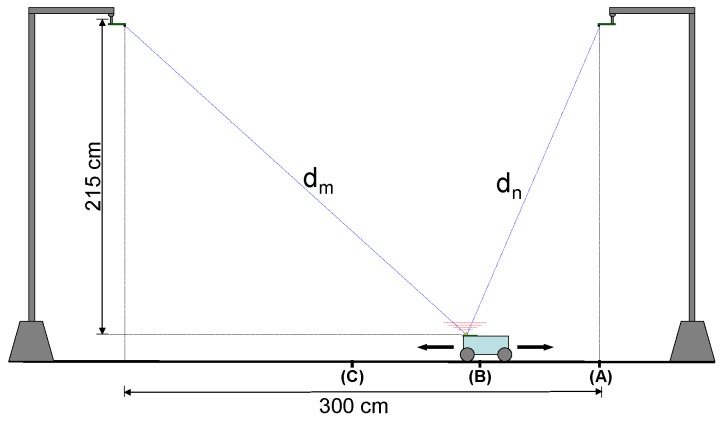
Setup for the real measurements.

Figure 11 shows the setup used to carry out the real measurements. The emitter is on board a mobile robot and two receivers are placed 2.15 m above the emitter and separated 3 m from each other. The robot was placed on three different locations (A,B and C in the figure) between both receivers, so that the combined SNR between both receivers would reach the desired levels. The minimum SNR case corresponds to the position (A), where the robot is directly under one of the receivers. The combined SNR in this case is 70 dB, dominated by the most distant receiver, given the high received power difference between both. The maximum SNR case correspond to the middle point between two receivers (C), where the individual SNR for every receiver is 93 dB, so the combined SNR is 90 dB.

In the particular case of the system that contextualizes this work, a maximum error has been defined for the application setup. The standard deviation of the differential distance error for this setup must be smaller than 5 cm for the worst SNR case (70 dB). Taking into account the propagation speed of the optical signal and the modulation frequency of 6 MHz, this distance error translates into a maximum standard deviation of the differential phase error of 6.3 mrad. Analyzing the results of the study and tests, sampling frequencies below 4.8 MHz would cause too much noise aliasing after the digitization, yielding unacceptable differential phase errors for the particular application constrains. Note that, given this error constraint, minimum acceptable sampling frequency is 2.5 times smaller than the minimum sampling frequency (12 MHz) to be used if, instead of band-pass sampling, standard low-pass sampling had been applied to digitize the signals. However, the error increment between these two cases is 1.4 times. Thus, sampling frequency is reduced by 60% at the cost of reducing precision by 29%.

## 6. Conclusions

A study on the relationship between sampling frequency and input SNR with final precision for a band-pass sampled differential phasemeter has been developed, considering the practical limitations, due to phase stability requirements, in the antialiasing filtering prior signals digitization.

The study was divided into two parts. In the first section, the reduction of signal-to-noise ratio around the signal band due to noise aliasing has been analyzed as a function of sampling frequency. This analysis demonstrates that the system design should consider the real parameters of the antialiasing filter used to reduce noise aliasing. A highly selective filter, efficient in reducing noise aliasing, shows a higher instability in its frequency response, causing a higher phase uncertainty. This instability depends on the drifts, mostly thermal, of the components of the filter. Due to this, quantifying the phase stability of the filter as a function of environmental conditions and particular features of its components, is a key task in the design of any phase measurement system. This becomes even more critical if undersampling is used, given its additional implications on the SNR degradation. The selection of the undersampling frequency ultimately depends on the filter features and the SNR degradation admissible by the application requirements. The method proposed in this section is independent from the technique used to extract the phase information from the digitized signals, establishing a general starting point for the design of undersampled phase measurement systems.

In the second part, the differential phase measuring architecture, based on asynchronous I/Q demodulation, is explained and the relationship between SNR of its input signals and achieved precision in the phase estimation is analyzed. An adequate approximation for the precision in the phase estimation depending on the input SNR of both signals is achieved. This study is validated by comparison between the theoretically estimated precision and results from real measurements using the explained architecture.

In the particular system that contextualizes this work, differential phases from IR signals are measured to estimate differential distances for indoor positioning applications. In this case, an undersampled digitization with the proposed measuring architecture allows for, compared to the minimum frequency of a low-pass sampling approach, a reduction of sampling speed by 60% while the loss of precision keeps below 29%. These results are achieved using a low-selectivity band-pass filter prior digitization, built with average thermal stability components and supporting a wide range of ambient temperature variation. The analyses carried out in this work allow the adequate selection, depending on the particular application requirements, of undersampling frequency, loss of precision and antialiasing filter specifications.

It can be concluded that, even if noise BW cannot be strictly reduced to prevent noise aliasing, applying band-pass sampling, with an adequate sampling frequency selection that takes into account the measuring architecture and the specifications of the analog system, allows for reduction of the digitizing system cost and the digital processing stage computing load with quantifiable and relatively reduced loss of precision.
